# Supplementary material for: Efficacy of an ultrasound training program for nurse midwives to assess high-risk conditions at labor triage in rural Uganda
Source: PLoS One. 2020 Jun 30;15(6):e0235269. doi: 10.1371/journal.pone.0235269 (PMC7326214; doi:10.1371/journal.pone.0235269)
Supplement: S1 File — (DOCX) [file pone.0235269.s001.docx]

**Date:_________**

**PTBI OB US Training: Final OSCE exam**

Participant Name:___________________

Observer (Examiner) Name:_________________

Time Start:________ Time End: _________ Total minutes for OSCE: ___________

|  | | **Answer** | |
| --- | --- | --- | --- |
|  |  | **Yes** | **No** |
| **A** | **COMMUNICATION AND INTERPERSONAL SKILLS** | | |
| 1 | Introduces self |  |  |
| 2 | Explains exam to patient and family |  |  |
| 3 | Demonstrates attention and modesty to the patient |  |  |
| **B** | **MACHINE SET UP** | | |
| 4 | Plug in machine with volt stabilizer |  |  |
| 5 | Correct machine placement (in line of site) |  |  |
| 6 | Correct amount of gel |  |  |
| 7 | Correct Probe |  |  |
| 8 | Correctly directs Probe Marker to patients right side or head |  |  |
| 9 | Correct Gain |  |  |
| 10 | Correct Depth |  |  |
| 11 | Correct hand positioning |  |  |
| 12 | Correctly chooses exam type of scanning (OB vs abdomen)? |  |  |
| 13 | Correctly chooses mode of scanning (B vs M mode)? |  |  |
| 14 | Understands language of tilting, rotating or sliding to describe movement of the probe |  |  |
| **C** | **SCAN: Labor Triage US** | | |
| 15 | Correct patient position for exam (side lying or flat with head up) |  |  |
| 16 | Correctly identifies Fetal heart |  |  |
| 17 | Correctly measures FHR using M Mode |  |  |
| 18 | Correctly identifies fetal head |  |  |
| 19 | Correctly describes fetal position |  |  |
| 20 | Correctly measures BPD |  |  |
| 21 | Correctly measures HC |  |  |
| 22 | Correctly measures FL |  |  |
| 23 | Correctly uses Report to generate Average GA |  |  |
| 24 | Correctly answers query regarding what measure to take if average GA is inconsistent or incorrect |  |  |
| 25 | Correctly measures TCD/CER |  |  |
| 26 | Correctly identifies number of intrauterine fetuses |  |  |
| 27 | Correctly identifies Placenta and location as anterior, posterior or fundal |  |  |
| 28 | Correctly identifies placental edge and follows placenta toward cervical os to assess for previa/low lying |  |  |
| 29 | Correctly looks for Amniotic fluid pockets in all four quadrants and identifies DVP |  |  |
| 30 | Correctly measures DVP and answers normal DVP measures question |  |  |
| **D** | **END EXAM** | | |
| 31 | Correctly explains to patient the findings of the ultrasound exam |  |  |
| 32 | Correctly documents exam in patient’s chart |  |  |
| 33 | Correctly documents exam for patient data sheet for study |  |  |
| 34 | Correctly cleans probe after use |  |  |
| **FINAL SCORE** | | **/34** | |

*Instructors please give feedback to participants after the test.*
